# HyReti-Net: hybrid retinal diseases classification and diagnosis network using optical coherence tomography

**DOI:** 10.3389/fmed.2025.1660920

**Published:** 2025-09-29

**Authors:** Jikun Yang, Chaoliang Hsu, Jing Wang, Bin Wu, Yuanyuan Lu, Yuxi Ding, Zhenbo Zhao, Kaili Tang, Feng Lu, Liwei Ma

**Affiliations:** ^1^Aier Eye Medical Center of Anhui Medical University, Hefei, Anhui, China; ^2^Shenyang Aier Excellence Eye Hospital, Shenyang, Liaoning, China; ^3^University of Southern California Translational Biotechnology Department, Los Angeles, CA, United States; ^4^School of Automation, Shenyang Aerospace University, Shenyang, Liaoning, China

**Keywords:** vision transformer, convolutional neural network, feature fusion, retinal diseases, classification

## Abstract

**Background:**

With optical coherence tomography (OCT), doctors are able to see cross-sections of the retinal layers and diagnose retinal diseases. Computer-aided diagnosis algorithms such as convolutional neural networks (CNNs) and vision Transformers (ViTs) enhance diagnostic efficiency by automatically analyzing these OCT images. However, CNNs are less effective in extracting global features and ViTs lack the local inductive bias and typically require large amounts of training data.

**Methods:**

In this paper, we presented a hybrid retinal diseases classification and diagnosis network named HyReti-Net which incorporated two branches. One branch extracted local features by leveraging the spatial hierarchy learning capabilities of ResNet-50, while the other branch was established based on Swin Transformer to consider the global information. In addition, we proposed a feature fusion module (FFM) consisting of a concatenation and residual block and the improved channel attention block to retain local and global features more effectively. The multi-level features fusion mechanism was used to further enhance the ability of global feature extraction.

**Results:**

Evaluation and comparison were used to show the advantage of the proposed architecture. Five metrics were applied to compare the performance of existing methods. Moreover, ablation studies were carried out to evaluate their effects on the foundational model. For each public dataset, heatmaps were also generated to enhance the interpretability of OCT image classification. The results underscored the effectiveness and advantage of the proposed method which achieved the highest classification accuracy.

**Conclusion:**

In this article, a hybrid multi-scale network model integrating dual-branches and a features fusion module was proposed to diagnose retinal diseases. The performance of the proposed method produced promising classification results. On the OCT-2014, OCT-2017 and OCT-C8, experimental results indicated that HyReti-Net achieved better performance than the state-of-the-art networks. This study can provide a reference for clinical diagnosis of ophthalmologists through artificial intelligence technology.

## 1 Introduction

The number of patients suffering from retinal diseases has increased significantly in recent years (1–3). Among the most prevalent retinal disease cases, age-related macular degeneration and diabetic macular edema are two common retinal diseases that are the leading cause of vision impairment and blindness (4). Therefore, automatic detection of retinal diseases has become a necessary procedure to produce accurate results for early diagnosis and timely treatment (5).

OCT serves as a vital imaging modality for the diagnosis of retinal lesions. This technique is distinguished by its non-invasive and contactless imaging capabilities, enabling the visualization of cross-sectional retinal layers and abnormalities (6–8). However, manual diagnosis of retinal OCT images presents several challenges (9–11). First, with the number of patients increasing annually, depending exclusively on qualified medical ophthalmologists is becoming insufficient to meet the growing diagnostic and therapeutic` demands. Secondly, certain lesions present with subtle features which are challenging to detect, increasing the risk of misinterpretation and diagnostic oversights (12–14).

Due to the progress in high-performance computing systems and deep learning algorithms, automatic diagnosis of retinal diseases has become a reality. Many researchers have made substantial advancements in the classification of OCT images through CNNs (15–21). However, in the analysis of retinal diseases, CNNs may focus more on local lesion features and fail to effectively extract global features. Therefore, it is necessary to integrate global retinal information with local lesion information to enhance classification performance.

Alternatively, advancements in natural language processing have catalyzed the progression of computer image processing from CNNs to Transformer-based sequence networks (22). Among them, ViT has emerged as the dominant architecture in computer vision (23). It is notable for its use of window-based self-attention to capture long-range dependencies in the whole image. However, utilizing a Transformer architecture exclusively may present difficulties in achieving adequate performance on small-scale datasets (13). Indeed, the Transformer technology inherently lacks local inductive biases and generally demands extensive training data. It poses a considerable hurdle in the retinal OCT image classification, where training images are scarce.

With the development of CNN and ViT, plenty of researchers have delved into the integration of CNNs with Transformers in the realm of image analysis tasks (24–26). On the basis of convolutional operations, CNNs excel at extracting local features and generating multi-scale feature information. Conversely, ViT utilizes a self-attention mechanism to effectively handle long-range and global dependencies in an image. The fusion of CNN and ViT promises to empower the model with enhanced robustness and efficiency in processing image information (12, 25, 27–30).

At present, the research gap in the classification of retinal diseases lay in the fact that traditional networks lacked multi-scale information and had limited ability to extract features from lesion areas, which reduced the classification accuracy of retinal diseases (31–33). To address the individual shortcomings of CNN and ViT, we proposed a hybrid model named HyReti-Net for classifying retinal diseases using OCT images. This model incorporated two branches: the CNN branch and the Transformer branch. Based on ResNet50, the CNN branch was responsible for extracting localized features and contextual information. Meanwhile, the Transformer branch, leveraging the Swin Transformer architecture, was tailored to capture global information and establish long-range dependencies. To evaluate the advantages of HyReti-Net model, we performed experiments on three public OCT datasets: OCT-2014 (34), OCT-2017 (35) and OCT-C8 (26).

The primary contributions of this research include:

1. We proposed HyReti-Net, a dual-branch architecture that seamlessly integrated CNN and Swin Transformer.
2. An innovative feature fusion approach was proposed, incorporating the concatenation and residual block and the improved channel attention block.
3. The muti-level features fusion mechanism was used to further enhance the ability of global feature extraction.
4. Grad-CAM method was used to improve the interpretability of the model and assist in confirming the accuracy of retinal diseases diagnosis.

## 2 Materials and methods

### 2.1 Data sources

Deep learning models had demonstrated exemplary performance on large-scale datasets (36). However, it was different to access to large and diverse datasets due to ethical concerns. Moreover, it was labor intensive and time consuming to obtain high-quality annotations for medical images. Consequently, we chose to use three existing OCT image datasets: OCT-2017, OCT-2014 and OCT-C8.

- OCT-2017 dataset consisted of 48,574 images in JPEG format divided into four categories: NORMAL, CNV, DME, and DRUSEN. This dataset was divided into training, testing, and validation folders for each image category.
- OCT-2014 dataset comprised a total of 3,231 SD-OCT collected from 45 patients. The whole dataset encompassed three distinct retinal conditions: 15 patients with normal retinal health, 15 patients with AMD, and 15 patients with DME.
- OCT-C8 dataset contained 24,000 images in JPG format divided into eight categories: Age-related Macular Degeneration (AMD), Central Serous Retinopathy (CSR), Choroidal Neo-Vascularization (CNV), Diabetic Macular Edema (DME), Diabetic Retinopathy (DR), DRUSEN, Macular Hole (MH), and NORMAL.

Based on the original data division strategy, both datasets were split image-wise into training set, validation set, and test set in a ratio of 7:2:1. The datasets information and distribution were listed in Table.1.

**Table 1:** Datasets information and distribution.

Datasets	Classes	Total	Training	Validation	Testing
OCT-2014	3	3,231	2,262	646	323
OCT-2017	4	48,574	34,002	9,715	4,857
OCT-C8	8	24,000	16,800	4,800	2,400

### 2.2 Experimental conditions

The proposed HyReti-Net network was trained and tested in PyTorch 3.10. The hardware of the experimental system included an NVIDIA GeForce RTX 3090 GPUan and an Intel Core i10 processor with 64 GB of RAM. By utilizing NVIDIA CUDA and its related parts, we observed substantial improvements in the convergence speed and overall efficiency. Based on grid search strategy, we selected the training hyperparameters for the proposed model. The Adam optimizer was utilized with a batch size of 128 and an initial learning rate of $1\times 10^{-4}$. The weight decay was set to 0.05, and the minimum learning rate was set to $1\times 10^{-6}$. The entire training period consisted of 100 epochs. During the training process, we adopted the the proposed loss function which combined focal loss and correlation loss to address the impact of class imbalance on classification tasks. The significance threshold p was set to 0.05. If the rate of change of adjacent errors is less than 0.5% in the training process, we consider that the training tended to stabilize. More experimental details of the training parameters were presented in Table 2.

**Table 2:** The detail of training parameters.

Parameter	Value	Parameter	Value
Epochs	100	Initial learning rate	0.0001
Batch Size	128	Minimum learning rate	0.000001
Optimizer	Adam	Significance threshold	0.05
Weight decay	0.05	Loss function	Hybrid loss function

### 2.3 Model design

**Figure 1:**
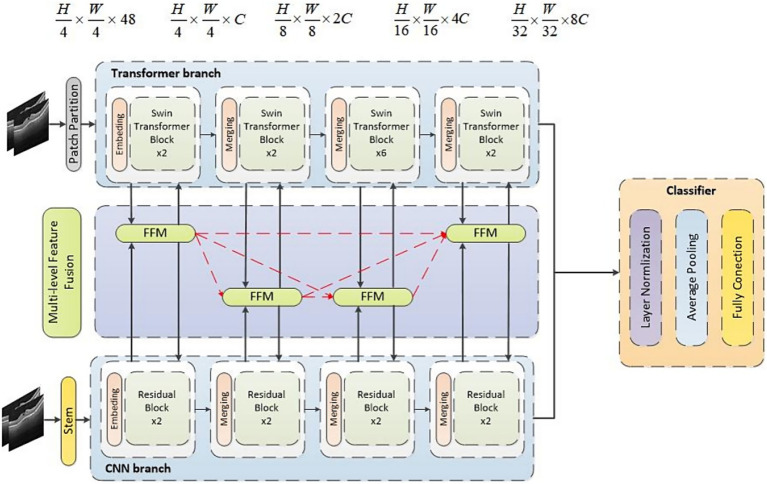
The architecture of HyReti-Net model.

The architecture of HyReti-Net model was shown in Figure 1. The overall structure consisted of four layers. Each layer encompassed multiple CNN and Swin Transformer blocks, accompanied by a feature fusion module. The CNN branch focused on extracting local features from OCT images, whereas the Transformer branch was responsible for capturing global information. The FFM further processed and refined the information from both branches, leveraging attention mechanisms to emphasize essential details and suppress unnecessary information.

While Swin Transformer captured global information, it primarily employed window-based multi-head self-attention, which was limited to local windows. Therefore, the muti-level features fusion mechanism was used to enhance the ability of global feature extraction. The fusion features would be transferred between FFMs. The whole process passed the fusion features of each layer to subsequent FFMs. Features from two FFMs would be added. By passing small-scale fusion features to large-scale fusion features, the feature presentations at different scales would be improved. The transmission direction among FFMs was shown by the red line in Figure 1. The muti-level features fusion mechanism enhanced the feature expression. The fused features were input into classifier for classification task. In the classifier, the fully connected layer flattened the output of the previous layer into one dimension to perform classification tasks. In this paper, the neurons in the fully connected layer were set to 1,024, followed by the Softmax activation function for retinal diseases diagnosis.

### 2.4 CNN branch

**Figure 2:**
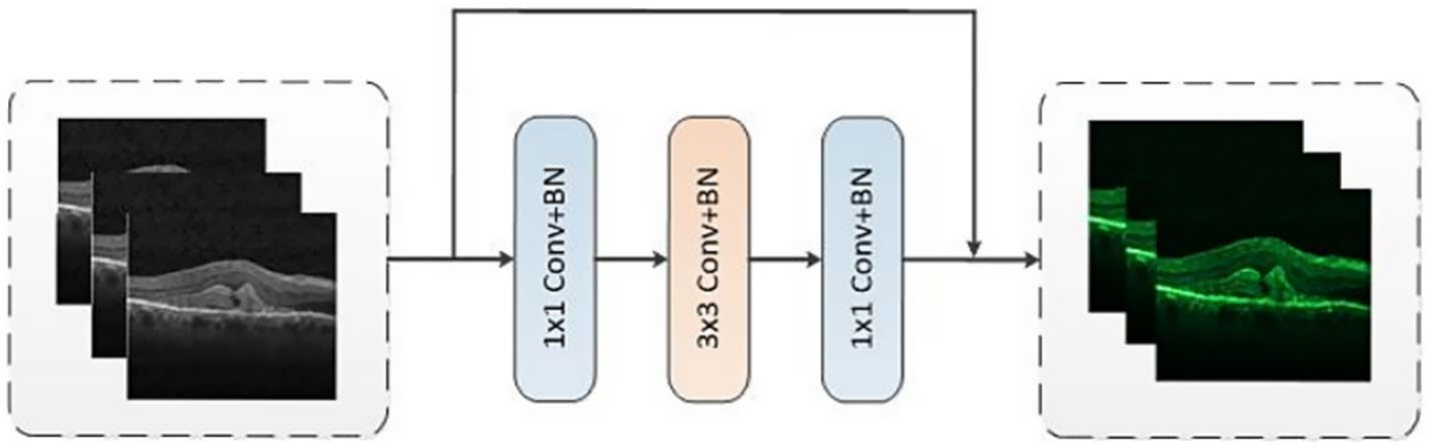
The structure of residual block.

We designed a CNN branch inspired by the ResNet50 architecture, with the primary objective of extracting both local features and contextual information from images. With the model depth increasing, the resolution progressively decreased while the number of channels increases. The overall structure comprised four layers and each layer consisted of two residual blocks, as shown in Figure 2. Each residual block was made up of three convolutional blocks: a 1×1 convolution for downsampling, a 3×3 convolution for spatial feature extraction, and a 1×1 convolution for upsampling. In addition, a residual module was incorporated to connect the input and output. CNN branch acquired local features by sliding convolutional kernels in the image, enabling them to more accurately capture local information, which added more refined local details into the features. To improve the training efficiency, the ResNet-50 backbone was trained on ImageNet (1). Then the backbone was fine-tuned from the pre-trained model based on the OCT classification tasks.

### 2.5 Transformer branch

**Figure 3:**
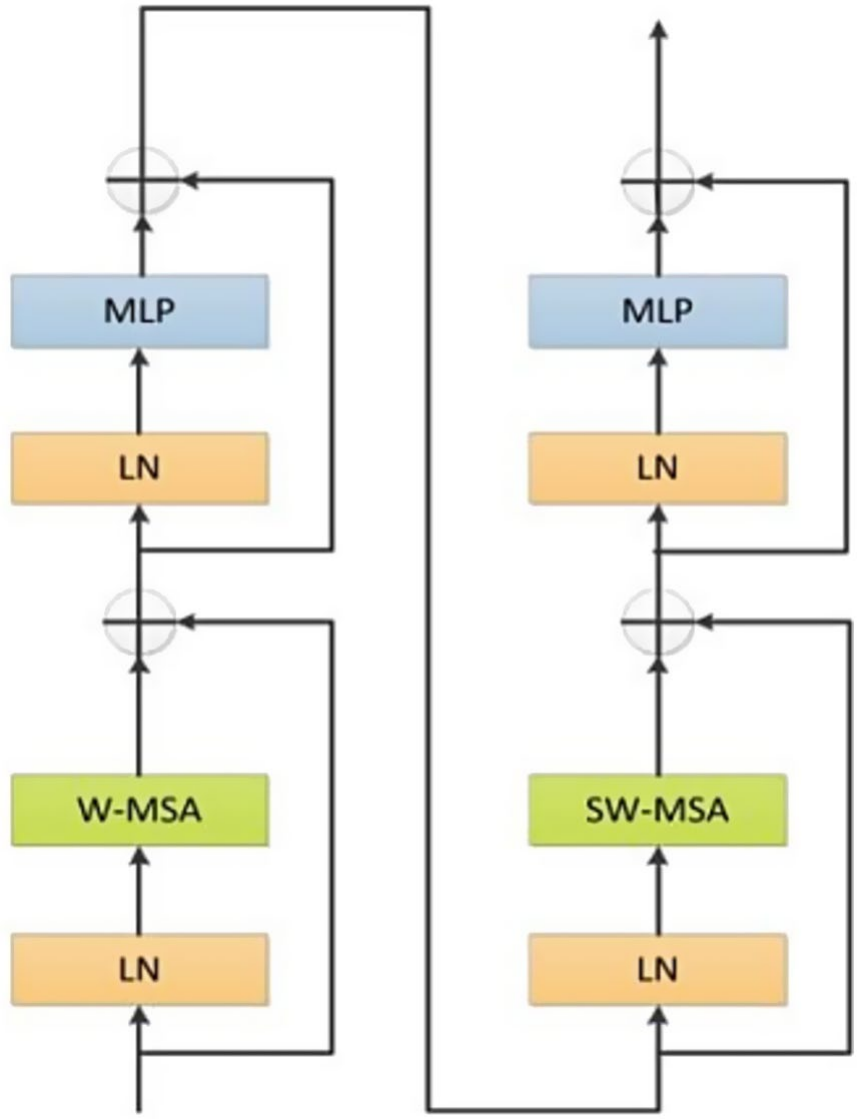
The structure of Swin Transformer.

In the Transformer branch, we utilized the original Swin Transformer architecture as the core framework which was accountable for extracting global information and long-range dependencies. Based on the application and analysis in Reference (13), the number of Swin Transformers was established as 2, 2, 6, and 2, respectively. The Swin Transformer module was shown in Figure 3.

Swin Transformer module was comprised of two sub-modules. The first sub-module comprised layer normalization, multi-layer perceptron and Window-Based Multi-Head Self-Attention (W-MSA). Meanwhile, the second sub-module was analogous but adopted a shifted W-MSA (SW-MSA). In addition, a patch merging module was utilized for the purpose of downsampling.

Given an input image, the proposed model employed the patch partition module in the first layer to divide the image into non-overlapping patches with fixed dimensions. Subsequently, these patches underwent a flattening process along the channel dimension. Between the second and fourth layers, the patch merging module was employed for downsampling. According to experimental results in Reference (26), the identical division strategy was executed. As Swin Transformer progressed features from the second to the fourth layers, the resolution was progressively reduced by half at each stage and the number of channels expanded to C, 2C, 4C and 8C, respectively.

**Figure 4:**
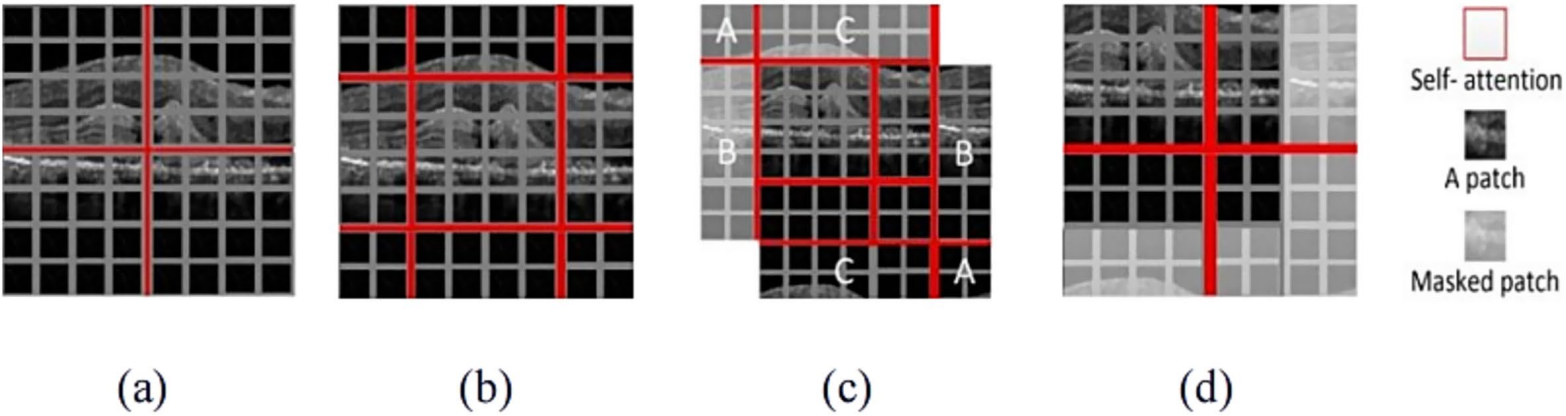
The illustration of W-MSA and SW-MSA. **(a)** The window partition based on W-MSA; **(b)** The shifting process based on SW-MSA; **(c)** Window cyclic process based on SW-MSA; **(d)** The results with masked patch.

Due to calculating relationships among all elements, W-MSA mechanism and SW-MSA mechanism were used in the Swin Transformer architecture. The principle of W-MSA and SW-MSA were shown in Figure 4.

W-MSA mechanism was used to split the whole image into patches with uniform size and applied attention separately to each patch, as shown in Figure 4a. When the W-MSA was utilized, the attention computation failed to establish cross-window connections, thereby constraining the ability to capture features of complex patterns. To address this limitation while maintaining the efficient computation of non-overlapping windows, SW-MSA mechanism was used, as illustrated in Figure 4b. Figure 4c demonstrated the cyclic shifting mechanism, which was designed to redistribute the image into four sections. The sections marked A, B and C were positioned in the top-left corner. Then, these sections underwent cyclic shifting to align with their respective corresponding regions. The splitting process resulted in an increasing number of windows, which demanded more computational resources and may introduce uncertainty when calculating attention mechanisms for non-contiguous regions. To address these limitations, a masking mechanism was proposed, as shown in Figure 4d. This mechanism was used to mask patches from various regions, allowing attention to be calculated only for patches in the boundaries of a single window. Through the shifting strategy, SW-MSA not only addressed the issue of limited interaction among different windows but also significantly decreased the computational burden, thereby enhancing overall computational efficiency.

### 2.6 Feature fusion module

**Figure 5:**
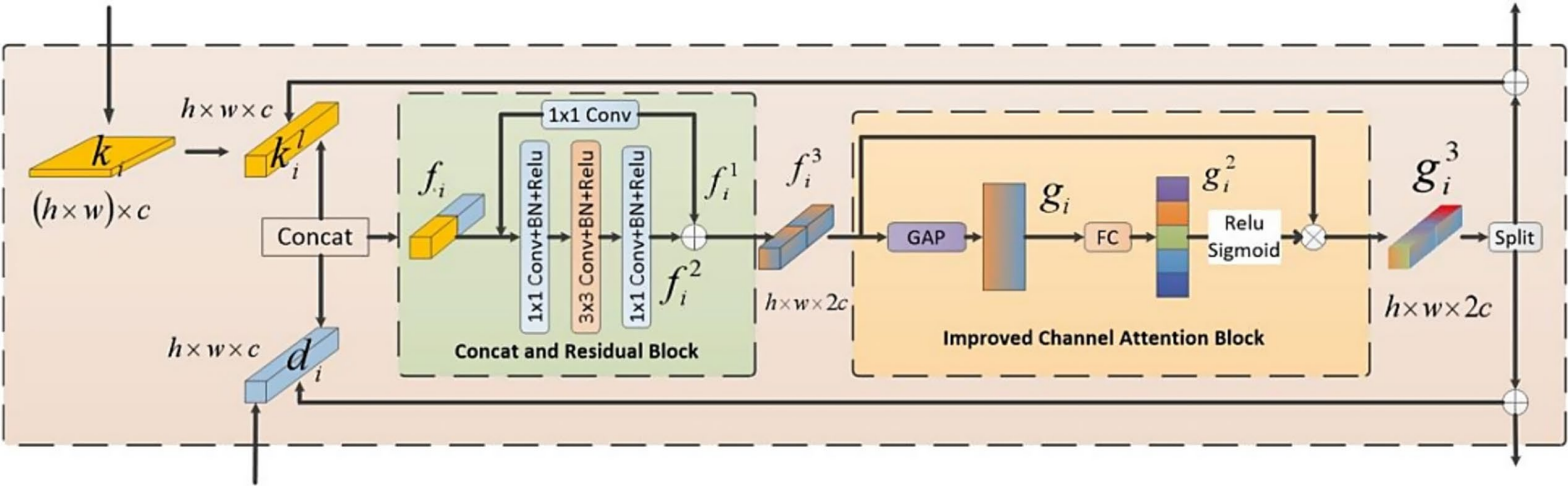
The structure of FFM.

Given the differing priorities in information extraction between the CNN and Swin Transformer branches, the combination of these two types of information was necessary. In order to seamlessly combine the local features extracted by the CNN branch with the global features captured by the Transformer branch, we proposed a FFM, as depicted in Figure 5.

The operational procedure of the FFM was outlined as follows: Firstly, we established bidirectional links for the features from both branches. Subsequently, a channel attention mechanism was utilized to emphasize critical features. Finally, the fused features were divided into two parts along the channel dimension, producing two components: $k_{i}$ and $d_{i}$ which denoted the feature maps originating from the Swin Transformer branch and the CNN branch, respectively. These refined features were then redirected to its corresponding branch and added to the original inputs, respectively.

The whole feature fusion module comprised two parts: the Concatenation and Residual Block (CRB) and the Improved Channel Attention Block (ICAB). CRB was employed to combine and extract features from both branches. First, a reshape operation was employed to change $k_{i}$ of dimension (h×w)×c into h×w×c. Subsequently, this module concatenated $d_{i}$ and $k_{i}$ along the channel to fuse features which was then fed into a residual structure for further learning. Another part was ICAB. Although different channels in the feature map encompassed diverse information, not all of their features were important. Our objective was to accentuate significant regions while disregarding irrelevant environmental factors. Therefore, we introduced an enhanced channel attention mechanism. First, we employed the global average pooling operation to reshape the fused features into a one-dimensional vector. Subsequently, this vector underwent a transformation through a fully connected layer to produce attention weights. Ultimately, these weights were used to compute a weighted matrix through a dot product operation between these attention weights and the input features. Along the channel dimension, we divided the fused feature map which were then redirected to its corresponding branch and added to the original inputs to continue training. In conclusion, FFM effectively integrated information from both branches. Consequently, the model was empowered to more effectively capture correlations among features.

### 2.7 Loss function

The imbalance of training data could affect the performance of the model. Inspired by Reference (37), we used a novel hybrid loss function in this paper to address the impact of class imbalance on classification tasks. The hybrid loss function combined focal loss and correlation loss.

1) Focal loss: Binary cross entropy was the theoretical basis of focal loss. Usually, binary cross entropy was defined as Equation 1:

(1) $$\begin{array}{l} L_{\mathrm{ce}}(\hat{y},y)=-y\log\hat{y}-(1-y)\log(1-\hat{y})\\ \;=\{\begin{array}{l} -\log\hat{y},\;\;y=1,\\ -\log(1-\hat{y}),\text{otherwise} \end{array} \end{array}$$

Where y represented the true label, and$\hat{y}$represented the predicted result of *y* = 1. For further derivation, the cross entropy could be defined as Equation 2:


(2) $$L_{\mathrm{ce}}(p)=-\log(p)$$

Where *p* was$\hat{y}$if *y* = 1. Otherwise, p was $1-\hat{y}$. On the basis of cross entropy, the focal loss function introduced a modulation factor ${\left(1-p\right)}^{\gamma }$, which reduced the weight of simple examples and made the training focus on difficult examples. Then the focal loss function could be defined as Equation 3:


(3) $$L_{\mathrm{fl}}(p)=-{\left(1-p\right)}^{\gamma }\log(p)$$

Where *γ* was the focusing parameter. The contribution of easy samples decreased with the increase of focusing parameters. In addition, the class imbalance problem could be solved by adding a weighting factor *α* to the focal loss. Therefore, the focal loss could be further redefined as Equation 4:


(4) $$L_{\mathrm{fl}}(\hat{y},y)=\{\begin{array}{l} -\alpha {\left(1-\hat{y}\right)}^{\gamma }\log(\hat{y}),\;\;y=1,\\ -(1-\alpha ){\hat{y}}^{\gamma }\log(1-\hat{y}),\;\;\;\text{otherwise}. \end{array}$$

2) Correntropy Loss: By adjusting the adaptability of sample distance through different L-norms, correntropy loss had better robustness against outliers. For very small errors, correlation had the property of L2 norm. As the error gradually increased, the correlation eventually reached the L0 norm. Based on the principle of Reference (37), the correlation entropy-induced loss function $L_{C}(\hat{y},y)$ could be defined as Equation 5:

(5) $$L_{C}(\hat{y},y)=1-k_{\sigma }(y-\hat{y})$$

Where $k_{\sigma }$represented the kernel function. In this paper, we took Gaussian kernel to calculate correntropy. The $L_{C}(\hat{y},y)$ could be expressed as Equation 6:


(6) $$\begin{array}{l} L_{C}(\hat{y},y)=1-G_{\sigma }(y-\hat{y})\\ \;=1-\exp(-{\left(y-\hat{y}\right)}^{2}/\sigma^{2}) \end{array}$$

3) The proposed loss function: By combining focal loss and correntropy loss, this paper proposed a hybrid loss function which was defined as Equation 7:

(7) $$L_{\mathrm{FC}}(\hat{y},y)=\{\begin{array}{l} L_{\mathrm{fl}}(\hat{y},y),\;\;\;0<t\leq n,\\ L_{C}(\hat{y},y),\;\;\;n<t\leq N \end{array}$$

Where *t* represented the current epoch and *n* represented the predefined threshold. In this paper, *n* was set to 30. HyReti-Net was trained for *n* epochs using focal loss and then correntropy loss was employed to complete the training. *N* referred to the total number of epochs. Correntropy loss had better generalization performance and robustness, but its non-convexity was prone to cause local minima. Then, we first used focal loss to pretrain the model to address the class imbalance problem.

## 3 Results

### 3.1 Evaluation method

In this study, we adopted accuracy (Acc), precision (Pre), recall (Rec), F1-score (FS) and specificity (Spe) as metrics for evaluating retinal diseases. The calculations of these evaluation metrics were based on the following formulas which were from Equations 8–12:


(8) $$\mathrm{Acc}=\frac{\mathrm{TP}+\mathrm{TN}}{\mathrm{TP}+\mathrm{TN}+\mathrm{FP}+\mathrm{FN}}$$

(9) $$\mathrm{Pre}=\frac{\mathrm{TP}}{\mathrm{TP}+\mathrm{FP}}$$

(10) $$\mathrm{Rec}/\text{Sensitivity}=\frac{\mathrm{TP}}{\mathrm{TP}+\mathrm{FN}}$$

(11) $$\mathrm{FS}=\frac{2\times \mathrm{Pre}\times \mathrm{Rec}}{\mathrm{Pre}+\mathrm{Rec}}$$

(12) $$\mathrm{Spe}=\frac{\mathrm{TN}}{\mathrm{TN}+\mathrm{FP}}$$

Where TP, TN, FP and FN represented true positive, true negative, false positive and false negative, respectively. Specifically, TP represented the number of correctly identified positive instances. TN represented the number of accurately identified negative instances in the model’s designated negative cases. FP denoted to the number of negative samples that were incorrectly classified as positive and FN represented the number of positive instances that were mistakenly labeled as negative.

### 3.2 Qualitative analysis

**Figure 6:**
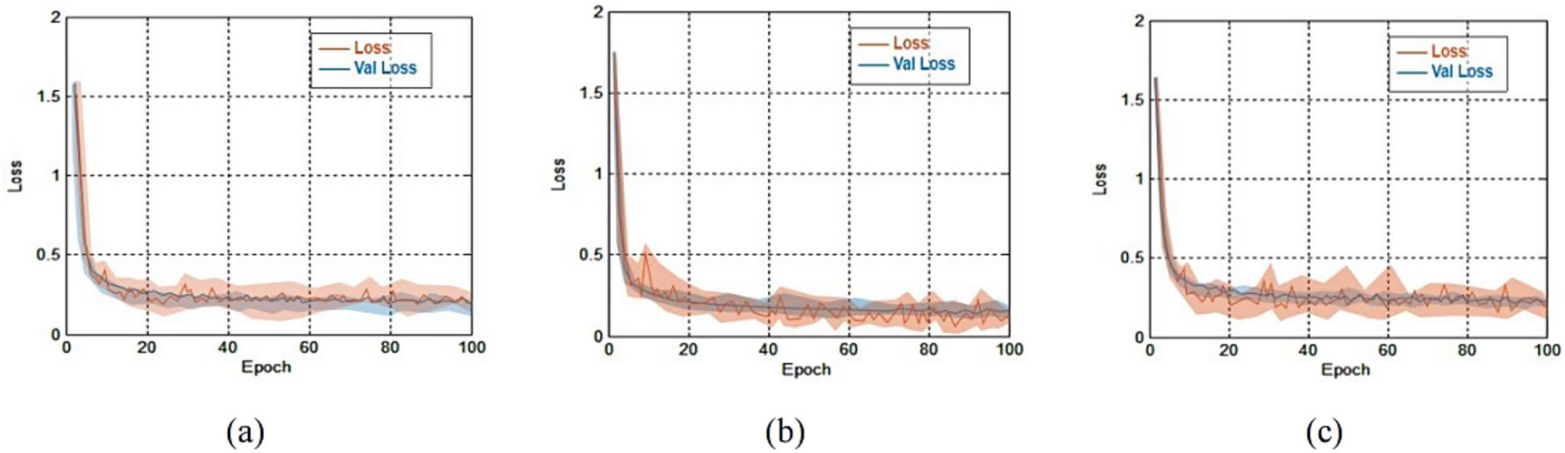
The errors comparison for both the training and validation sets on three public datasets. **(a)** Loss comparison on OCT-2014 and the average error was 0.41 ± 0.22; **(b)** loss comparison on OCT-2017 and the average error was 0.45 ± 0.28; **(c)** loss comparison on OCT-C8 and the average error was 0.49 ± 0.33.

In this experiment, we systematically tracked the loss error for both the training and validation sets. The recorded values served as the basis for evaluating the model classification capabilities. The error progression for both the training and validation sets on three public datasets were shown in Figure 6.

Results from Figure 6 shown that training errors and validation errors rapidly decreased in the initial stage. If the rate of change of adjacent errors was less than 0.5%, we considered that the training tended to stabilize. On the OCT-2014, OCT-2017 and OCT-C8 databases, the minimum errors lay in the 38th epoch, the 51st epoch and the 60th epoch, respectively. On the OCT-2014, OCT-2017 and OCT-C8 databases, the average errors were 0.41 ± 0.22, 0.45 ± 0.28, and 0.49 ± 0.33, respectively. Due to similar trends and small errors observed between the training and validation processes, the model demonstrated that there were no signs of overfitting.

### 3.3 Quantitative analysis

**Figure 7:**
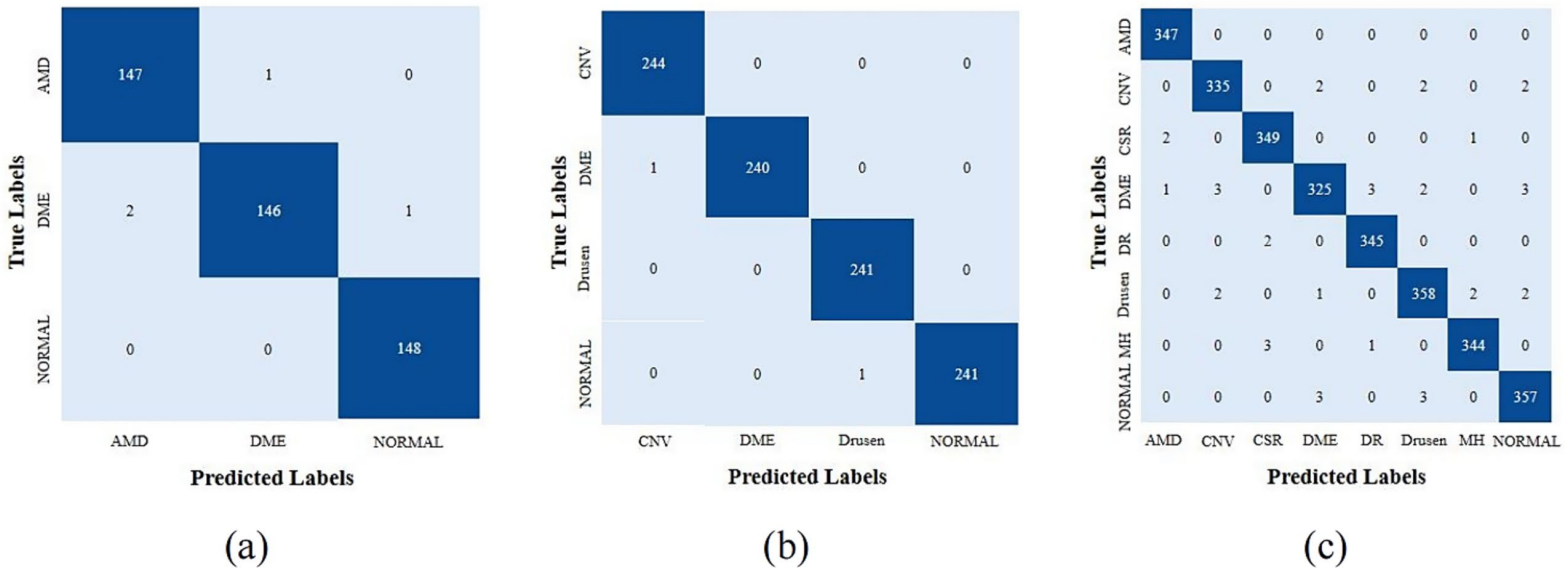
Confusion matrices of different datasets generated by HyReti-Net. **(a)** Confusion matrices from OCT-2014; **(b)** Confusion matrices from OCT-2017; **(c)** Confusion matrices from OCT-C8.

In order to accurately and quantitatively analyze the performance of the model, we first provided the confusion matrices that were generated by HyReti-Net. The confusion matrices shown that HyReti-Net was capable of accurately classifying and detecting retinal diseases, as shown in Figure 7. Results from confusion matrices verified the feasibility of the proposed model.

In quantitative analysis, the performance of the network was evaluated and analyzed utilizing the five specific metrics: Acc, Pre, Rec, Fs and Spe. To increase the generalizability of the model, we provided cross-validation with 3-fold on three public datasets and the average value served as the final value. The detailed classification metrics on the test dataset was presented in Table 3.

**Table 3:** The detailed evaluation metrics of the HyReti-Net model.

Dataset	Class	Evaluation metrics(%)				
		Acc	Pre	Rec	FS	Spe
OCT-2017	CNV	99.62	99.33	99.31	99.32	98.26
	DME	99.64	98.99	99.73	99.36	99.68
	DRUSEN	98.25	99.37	99.44	99.40	99.52
	NORMAL	99.66	99.05	99.21	99.13	99.27
	Average	99.29	99.19	99.42	99.30	99.18
OCT-2014	AMD	99.25	98.92	99.76	99.34	99.26
	DME	98.32	99.82	98.96	99.39	99.44
	NORMAL	99.76	99.63	99.22	99.42	99.27
	Average	99.11	99.46	99.31	99.38	99.32
OCT-8	AMD	99.14	98.41	99.27	97.58	97.35
	CNV	98.55	98.37	97.76	98.44	97.43
	CSR	98.44	97.46	98.16	98.37	98.34
	DME	98.13	98.19	98.23	99.24	98.16
	DR	98.85	97.46	99.34	97.95	98.47
	DRUSEN	98.08	97.55	98.46	98.43	97.38
	MH	99.13	98.86	99.37	98.76	98.25
	NORMAL	98.07	98.91	98.46	98.14	98.71
	Average	98.55	98.15	98.63	98.36	98.01

Based on these results, HyReti-Net exhibited good performance in terms of five specific metrics. Specifically, in the OCT-2017 and OCT-2014 datasets, the model exhibited remarkable performance in the classification of AMD, DME, and Normal cases with average accuracy of 99.29 and 99.11%, respectively. In the OCT-C8 dataset, HyReti-Net achieves an average accuracy of 98.55% for eight distinct cases. Data analysis shown that the HyReti-Net has high level of accuracy in identifying fundus diseases. This achievement highlights the model’s remarkable predictive performance.

### 3.4 Features map visualization

**Figure 8:**
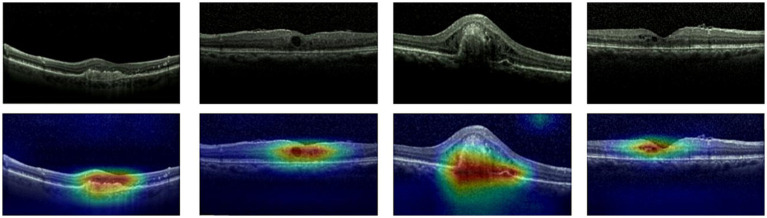
Features map visualization of HyReti-Net on OCT-2014 dataset. The first line is the original OCT images. The second line is class activation mapping visualizations based on Grad-CAM method.

**Figure 9:**
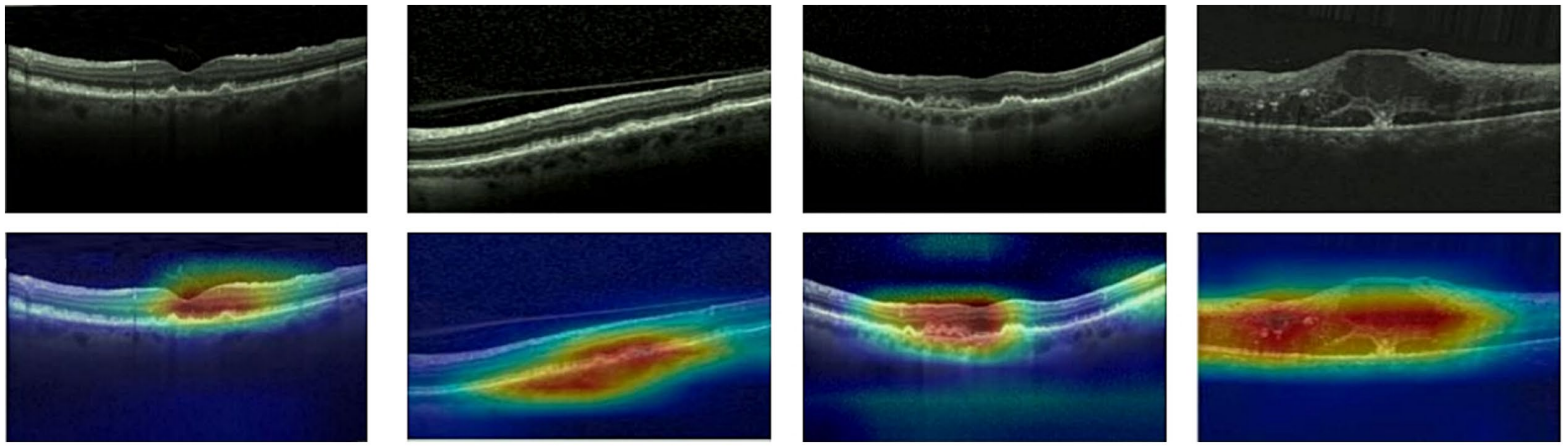
Features map visualization of HyReti-Net on OCT-2017 dataset. The first line is the original OCT images. The second line is class activation mapping visualizations based on Grad-CAM method.

**Figure 10:**
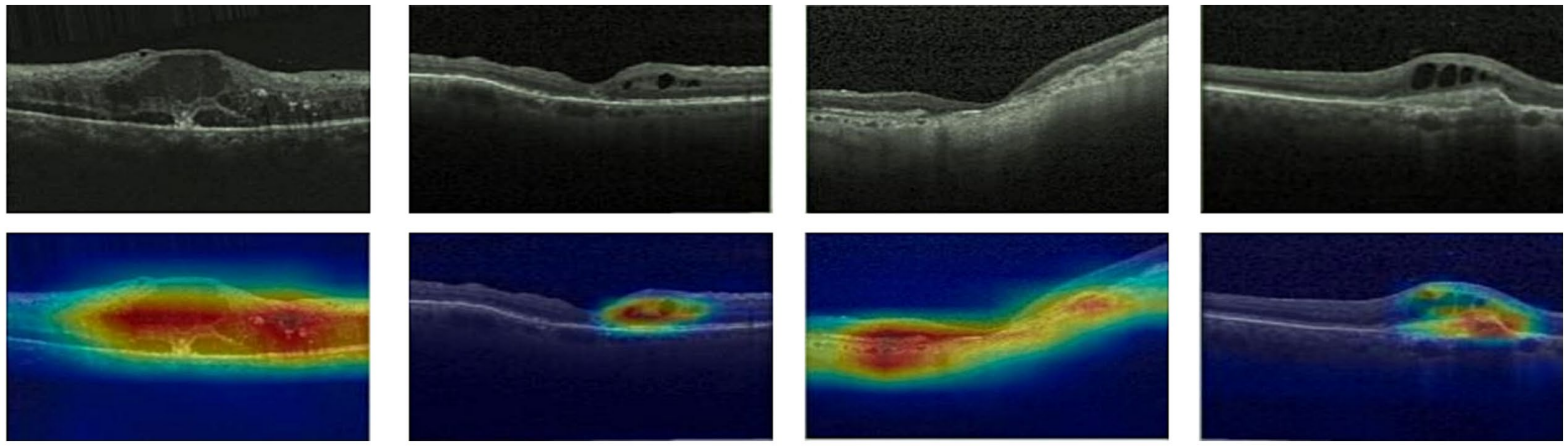
Features map visualization of HyReti-Net on OCT-C8 dataset. The first line is the original OCT images. The second line is class activation mapping visualizations based on Grad-CAM method.

Given ethical considerations and direct impact on human life and health, the interpretability of deep learning models could help clinicians, patients, and researchers in comprehending, trusting, and efficiently employing artificial intelligence technologies in the healthcare domain. In this section, we utilized the Grad-CAM method (38) to present Class Activation Mapping (CAM) visualizations, which could demonstrate the evidence underlying the predictions of the proposed model. The heat maps were generated by the CAM technique to emphasizes specific regions in an OCT image that were closely linked to the target class. The Grad-CAM showed the regions of interest. As shown in Figures 8, 9, 10, the Grad-CAM were generated based on original OCT images. Deeper red color represented stronger correlation with the predicted category. From the Grad-CAM, we found that the lesion regions were all appear with red. It demonstrated that the model payed attention to the crucial regions, which aligned with the diagnostic process of ophthalmologists.

### 3.5 Validation and comparative analysis

To further confirm the efficacy of the HyReti-Net model, we compared it with several widely adopted classification models. The comparative results on three public OCT datasets were presented in Table 4.

**Table 4:** The comparative results on three public OCT datasets.

Datasets	Models	Evaluation metrices (%)				
		Accuracy	Sensitivity	Precision	F1-Score	Specificity
OCT-2017	ResNet50 (3)	92.23	94.06	93.42	93.74	92.16
	ViT (25)	93.19	93.28	92.37	92.82	93.24
	HTCNet (Hybrid) (28)	97.96	96.25	97.88	97.06	96.53
	WaveNet-SF (Hybrid) (29)	98.14	98.22	97.82	98.02	97.61
	MSLl-Net (Hybrid) (30)	98.57	98.46	97.88	98.17	97.36
	HyReti-Net (proposed)	99.06	99.12	98.98	99.05	98.08
OCT-2014	ResNet50 (3)	94.89	94.35	92.77	93.55	94.82
	ViT (25)	90.74	92.59	90.63	91.60	94.73
	HTCNet (Hybrid) (28)	97.85	97.24	96.55	96.89	97.31
	WaveNet-SF (Hybrid) (29)	98.36	98.39	98.22	98.30	98.42
	MSLl-Net (Hybrid) (30)	98.27	98.59	98.03	98.31	98.38
	HyReti-Net (proposed)	99.44	99.08	99.25	99.16	99.07
OCT-C8	ResNet50 (3)	93.92	91.27	91.05	92.54	93.15
	ViT (25)	88.43	90.36	89.25	90.13	93.25
	HTCNet (Hybrid) (28)	95.33	96.13	94.76	94.34	96.28
	WaveNet-SF (Hybrid) (29)	97.63	97.04	97.05	97.23	97.22
	MSLl-Net (Hybrid) (30)	96.39	96.62	96.46	96.45	96.49
	HyReti-Net (proposed)	98.76	97.73	98.43	98.33	97.95

Through analysis of different models, the HyReti-Net model consistently showed superior results, outperforming other selected models on three datasets. As illustrated in Table 4, HyReti-Net achieved accuracy of 99.06, 99.44 and 98.76%, with F1-Score values of 99.05, 99.16 and 98.33% on the OCT-2017, OCT-2014 and OCT-C8.

To further evaluate the proposed model under limited training data scenarios, we only used one-tenth images from OCT-2014 to build the new database. Then the new dataset had a total of 323 images which consisted of 214 training images, 65 validation images and 44 test images. To reduce the impact of imbalance on classification results, we ensured that the number of images in each category was equal. We compared the performance of different models on the new dataset. The comparison results were shown in Table 5.

**Table 5:** The comparative results on the small OCT dataste.

Models	Evaluation metrices (%)				
	Accuracy	Sensitivity	Precision	F1-Score	Specificity
ResNet50 (3)	86.35	85.17	83.25	85.46	84.47
ViT (25)	82.27	83.39	81.56	82.17	83.34
HTCNet (Hybrid) (28)	88.66	87.26	84.35	83.39	85.19
WaveNet-SF (Hybrid) (29)	89.37	88.34	84.26	84.47	88.26
MSLl-Net (Hybrid) (30)	87.18	88.39	85.33	86.63	87.48
HyReti-Net (proposed)	92.36	91.54	88.37	89.35	91.16

The comparison results demonstrated that CNN was more suitable than ViT for classification tasks on small datasets. The results exhibited the shortcomings of ViT, which accorded with the conclusion from Reference (36). Due to integrating the local features and global features relationship, hybrid models overcame ResNet50 and ViT. Compare the HTCNet, WaveNet-SF and MSLl-Net, HyReti-Net could achieve the best performance on the new dataset, which demonstrated that HyReti-Net was able to achieve higher classification accuracy than other hybrid models under limited training data scenarios.

### 3.6 Ablation study

In this study, we proposed HyReti-Net model with dual-branch structure and FFM module. To evaluate the effectiveness of HyReti-Net, we applied various modifications to the model. Acc, Pre, Rec, and FS were also used as the metrics for evaluation. Eight schemes were tested on the three public datasets and the average values were set as the final values. Scheme 0 was the baseline model which combined ResNet and Transformer. The features from both branches were concatenated directly. Scheme 1 and Scheme 2 employed ResNet50 and Swin architectures, respectively. Scheme 3 combined two ResNet50 architectures to generate a simple hybrid model which were concatenated directly instead of using CRB and ICAB. Scheme 4 was similar to Scheme 3, which combined two Transformer architectures. On the basis of the dual-branch structure, the ICA and CBR modules were, respectively, incorporated into the basic model to generate Scheme 5 and Scheme 6. Scheme 7 represented the proposed HyReti-Net model. The performance of above eight schemes were presented in Table 6.

**Table 6:** The performance of eight schemes.

Schemes	Networks	Acc	Pre	Rec	FS
Scheme0	ResNet+Transformer	94.08%	96.73%	95.53%	96.23%
Scheme1	ResNet	92.77%	94.16%	93.75%	93.68%
Scheme2	Transformer	93.58%	95.57%	93.33%	95.52%
Scheme3	ResNet+ResNet	93.57%	96.06%	94.37%	95.83%
Scheme4	Transformer+Transformer	92.49%	95.58%	93.86%	93.47%
Scheme5	ResNet+Transformer+CBR	94.35%	97.73%	96.14%	97.27%
Scheme6	ResNet+Transformer+ICAB	96.26%	97.98%	96.89%	97.82%
Scheme7	ResNet+Transformer+CBR + ICAB	99.25%	99.10%	99.12%	99.11%

By comparing results from Scheme 0, Scheme 1 and Scheme 2, Scheme 0 has better performance. It meat that the fusion of local features and global features could achieve better classification results than that from a single type of feature. Compared with Scheme 3 and Scheme 4, Scheme 0 has better performance. It demonstrated that multi-level feature fusion methods could improve the classification performance. From Scheme 0 to Scheme 4, all networks isolated the impact of CRB and ICAB. Building upon the dual-branch model, Scheme 5 incorporated the CRB attention mechanism, resulting in substantial improvements in Acc, Pre, Rec and FS. This also underscored a remarkable enhancement in performance attributed to the integration of the attention mechanism. Scheme 6 incorporated the ICAB module into the dual-branch model, producing further performance improvements. Specifically, compared to Scheme 5, there was an increase of 1.91% in accuracy, 0.25% in precision, 0.75% in recall, and 0.55% in FS. Ultimately, Scheme 7 incorporated both the dual-branch structure and the FFM module, achieving the highest values for all metrics: 99.25% Acc, 99.10% Pre, 99.12% Rec, and 99.11% FS. These results demonstrated the efficacy of the proposed HyReti-Net model in improving the overall performance of the model.

## 4 Discussion

The primary objective of our study was to develop a hybrid model named HyReti-Net for the accurate classification of retinal diseases. Inspired from many recent studies (29–33), HyReti-Net contained a dual-branch architecture for feature extraction and a FFM. Due to leveraging the strengths of both paradigms, HyReti-Net was able to capture both local features via the CNN and global information with long-range dependencies through the Transformer. Moreover FFM was designed to merge these two types of information, thereby enabling the model generating more comprehensive features for classifying OCT scans. Three different datasets were used to validate the performance and generalization of HyReti-Net.

Experimental results indicated that HyReti-Net achieved accuracy of 99.06, 99.44 and 98.76%, with F1-Score values of 99.05, 99.16 and 98.33% on the OCT-2017, OCT-2014 and OCT-C8, respectively. Compared to other state-of-the-art models (29–33), the performance of HyReti-Net was superior to traditional models and hybrid approaches. On the OCT-2017 dataset, HyReti-Net notably exceeded HTCNet, WaveNet-SF and MSLl-Net by 1.1, 0.92, and 0.49 percentage points in accuracy, respectively. On the OCT-2014 dataset, HyReti-Net exceeded HTCNet, WaveNet-SF and MSLl-Net by 1.59, 1.08, and 1.17 percentage points in accuracy, respectively. Similarly, on the OCT-C8 dataset, HyReti-Net exceeded HTCNet, WaveNet-SF and MSLl-Net by 3.43, 1.13 and 2.37 percentage points in accuracy, respectively. The comparative results underscored the effectiveness and advantage of HyReti-Net. These results from HyReti-Net underscored its robustness and superiority. Through analysis of the experimental results, we were also able to find that classes with limited sample sizes exhibited comparatively lower precision and recall in the classification results. In addition, we also evaluated and compared HyReti-Net with several classic methods. The results underscored the effectiveness and advantage of HyReti-Net which achieved the highest classification accuracy.

In addition, a FFM module was proposed in this paper. The whole module consisted of CRB and ICAB. CRB was employed to combine and extracted features from both branches. Then ICAB was utilized to emphasize critical features based on channel attention mechanism. The proposed FFM module was different from traditional feature fusion mechanism (29–32). Along the channel dimension, we divided the fused feature map which was then redirected to its corresponding branch and added to the original inputs to continue training iteratively. Therefore the model could achieve superior performance. Ablation studies were carried out to evaluate their effects on the foundational model, as presented in Table 6. Comparison results further demonstrated the advantages of FFM. In comparison to using single ResNet50 or Swin Transformer branch, the dual-branch structure with ICAB attention mechanism exhibited significant improvements in Acc, Pre, Rec, and FS. Moreover, the dual-branch structure with the FFM module could achieve the best performance. The FFM module has a very high repeatability. Similar to the traditional feature concatenation process, the FFM module is designed to fuse the features from two branches. The overall structure still adopts basic structures such as convolution, pooling and residuals. The FFM module simultaneously introduces the channel attention mechanism. By introducing different attention mechanisms and fusion forms, the FFM module has strong scalability and computational efficiency.

For each public dataset, heatmaps were also generated to enhance the interpretability of OCT image classification, as shown in Figures 8, 9, 10. The heatmaps showed the specific regions in an OCT image that were closely linked to the target class. It meant that HyReti-Net was able to accurately focus on the lesion area, which meant that the corresponding classification performance was better.

Although our proposed hybrid model had demonstrated successes, this study also had some limitations. First, due to the challenges in acquiring medical images and time limitations, HyReti-Net was trained and tested on the public datasets instead of clinical datasets. Second, we only explored dual-branches structure: CNN branch and Transformer branch. Multiple-branches structure could be further explored based on HyReti-Net. Third, we only used OCT images in this paper, without studying the performance of multimodal images. In the future, we will conduct research using clinical retinal images and more effective feature extraction methods. Additionally, we will focus on the application of multimodal images and external fundus diseases to verify the generalization ability and robustness of the model. The computational complexity analysis, including model size, parameter counts, inference speed, and training time will be used to evaluate the practical utility of the proposed model.

## 5 Conclusion

Retinal diseases can lead to temporary or permanent visual impairments. In this study, we proposed a hybrid multi-scale network model, named HyReti-Net, integrating dual-branches and a features fusion module. One branch utilized ResNet50 to extract local features while the other branch employed the Swin Transformer to capture global information and long-range dependencies. Finally, local and global features were fused in the feature fusion module. When evaluated on three public retinal datasets, HyReti-Net achieved accuracy of 99.06% in the four-category task, accuracy of 99.44% in the three-category task and accuracy of 98.76% in the eight-category task. Additionally, we used the Grad-CAM method to generate heat maps to improve the interpretability of the model classification results. This study can provide a reference for clinical diagnosis of ophthalmologists through artificial intelligence technology. Moreover, it helps improve the accuracy of retinal disease diagnosis, and it will play an important role in preventing blindness caused by retinal diseases.

## Data Availability

The original contributions presented in the study are included in the article/supplementary material, further inquiries can be directed to the corresponding author.
